# Supplementary figures and images for: Control of Gene Expression by Proteins That Bind Many Alternative Nucleic Acid Structures Through the Same Domain
Source: Int J Mol Sci. 2025 Dec 26;27(1):272. doi: 10.3390/ijms27010272 (PMC12785367; doi:10.3390/ijms27010272)

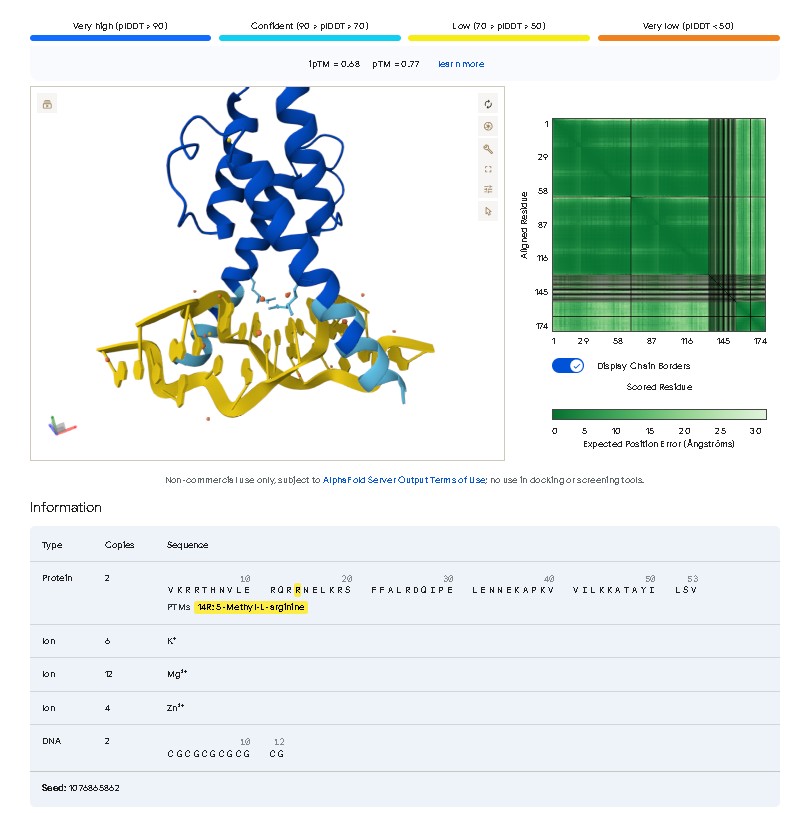

Supplement: Supplementary file 1 [file ijms-27-00272-s001.zip › AlphaFold V3 screenshot c-MYC-Z-DNA.jpg]
